# Fascioliasis risk factors and space-time clusters in domestic ruminants in Bangladesh

**DOI:** 10.1186/s13071-017-2168-7

**Published:** 2017-05-08

**Authors:** A. K. M. Anisur Rahman, SK Shaheenur Islam, Md. Hasanuzzaman Talukder, Md. Kumrul Hassan, Navneet K. Dhand, Michael P. Ward

**Affiliations:** 1 Department of Medicine, Bangladesh Agricultural University, Mymensingh, 2202 Bangladesh; 2 Department of Livestock Services, Krishi Khamar Sarak, Farmgate, Dhaka, 1215 Bangladesh; 3 Department of Parasitology, Bangladesh Agricultural University, Mymensingh, 2202 Bangladesh; 4 Sydney School of Veterinary Science, The University of Sydney, Camden, 2570 NSW, Australia

**Keywords:** Fascioliasis, Domestic ruminants, Hot-spots, Space-time clusters, Risk factors, Bangladesh

## Abstract

**Background:**

A retrospective observational study was conducted to identify fascioliasis hotspots, clusters, potential risk factors and to map fascioliasis risk in domestic ruminants in Bangladesh. Cases of fascioliasis in cattle, buffalo, sheep and goats from all districts in Bangladesh between 2011 and 2013 were identified via secondary surveillance data from the Department of Livestock Services’ Epidemiology Unit. From each case report, date of report, species affected and district data were extracted. The total number of domestic ruminants in each district was used to calculate fascioliasis cases per ten thousand animals at risk per district, and this was used for cluster and hotspot analysis. Clustering was assessed with Moran’s spatial autocorrelation statistic, hotspots with the local indicator of spatial association (LISA) statistic and space-time clusters with the scan statistic (Poisson model). The association between district fascioliasis prevalence and climate (temperature, precipitation), elevation, land cover and water bodies was investigated using a spatial regression model.

**Results:**

A total of 1,723,971 cases of fascioliasis were reported in the three-year study period in cattle (1,164,560), goats (424,314), buffalo (88,924) and sheep (46,173). A total of nine hotspots were identified; one of these persisted in each of the three years. Only two local clusters were found. Five space-time clusters located within 22 districts were also identified. Annual risk maps of fascioliasis cases correlated with the hotspots and clusters detected. Cultivated and managed (*P* < 0.001) and artificial surface (*P* = 0.04) land cover areas, and elevation (*P* = 0.003) were positively and negatively associated with fascioliasis in Bangladesh, respectively.

**Conclusions:**

Results indicate that due to land use characteristics some areas of Bangladesh are at greater risk of fascioliasis. The potential risk factors, hot spots and clusters identified in this study can be used to guide science-based treatment and control decisions for fascioliasis in Bangladesh and in other similar geo-climatic zones throughout the world.

## Background

Fascioliasis, caused by *Fasciola gigantica*, is endemic in domestic ruminants in Bangladesh [1]. The disease causes considerable economic impact due to mortality, liver condemnation, reduced weight gain (up to 20%) and reduced quality and quantity (3–15% loss) of milk production [2, 3]. Globally, more than 700 million domestic ruminants are at risk and economic loss exceeds US$ 3 billion per year [3]. Human fascioliasis is considered as a neglected tropical disease [4] affecting approximately 50 million people worldwide [5].

The prevalence of fascioliasis in Bangladesh in live animals has been reported to vary from 21 to 53% in cattle [6–16], 10 to 32% in goats [15–19], 8.4 to 31% in sheep [16, 18, 20] and 19 to 51% in buffaloes [15, 16, 21–27]. The prevalence of fascioliasis in slaughtered animals has been reported to be 15–66% in cattle [14, 28], 3.8–22% in goats [17, 29–31], 81% in sheep [32] and 23–47% in buffaloes [22, 33], respectively. The actual burden of fascioliasis - including subclinical disease - is likely much higher than that reported above [34].

Some demographic and seasonal risk factors for fascioliasis in domestic ruminants have also been reported [6, 11, 13, 17, 30, 35]. The epidemiology of fascioliasis has a spatial element due to the free-living stages of *F. giantica* - as well as the intermediate snail host *Lymnaea auricularia* var. *rufescens* [36, 37] - and the influence of climatic and environmental conditions [38]. No study on the spatial distribution of fascioliasis in domestic ruminants in Bangladesh based on national level data has been published. Understanding the spatial distribution of this disease and identifying clusters, hotspots, risk factors and risk mapping are vital to focus scarce resources for treatment and control programs on the most at-risk areas. The objectives of this study were to identify high risk areas and potential fascioliasis risk factors for domestic ruminants in Bangladesh.

## Methods

### Data

#### Fascioliasis case data

Bangladesh consists of 64 districts and 489 sub-districts (*upazila*). In every *upazila* there is a veterinary hospital. Cases attending these hospitals are recorded, which is the only disease surveillance system in use in Bangladesh. Sub-districts report monthly surveillance data to districts which compile and forward data to the Epidemiology Unit of the Department of Livestock Service (DLS). Fascioliasis records in cattle, goats, sheep and buffalo during 2011 to 2013 were sourced from DLS and used for this study. These cases were diagnosed based on the direct fecal smear test following standard procedures [39]. In brief, fresh fecal samples were collected from the rectum of individual cattle in dry, clean polythene bags. At least three smears were prepared for each sample and *Fasciola* eggs were identified on the basis of their morphological features. The data included information on case date, sub-district and district name, species (cattle, goat, sheep or buffalo), and number of deaths due to fascioliasis; number of species of domestic ruminants in each district was also available from DLS.

#### Climate, elevation, inland water and land cover data

The ESRI grid format average temperature (°C × 10) and precipitation (mm) data (22 km^2^ resolution) were downloaded from the global climate data (www.worldclim.org). These data were converted to ESRI shape files using DIVA-GIS 7.5 (www.diva-gis.org) software. The Bangladesh district-specific monthly temperature and precipitation data were then obtained through a spatial join with a Bangladesh district map attribute table in ArcGIS 10.3.1. The monthly temperature and precipitation records per district were aggregated to four seasons: winter (December to February), pre-monsoon (March to May), monsoon (June to August) and post-monsoon (September to November) [40]. The inland water, country mask elevation and land-cover data were downloaded from the DIVA-GIS website (www.diva-gis.org). These elevation and land-cover data were read and Bangladesh district-level values were calculated in ArcGIS 10.3.1 using a spatial join. The length of river (polyline) and area of water bodies (polygon) per district were calculated by adding a respective field in the attribute table. The Bangladesh district shape file containing annual number fascioliasis cases, total number of fascioliasis cases, log-transferred total fascioliasis cases, elevation, land-cover, river length, area of water bodies, seasonal temperature and precipitation data were used for the spatial regression analysis.

### Analysis

#### Descriptive statistics

Fascioliasis case records were entered into Microsoft Excel®. Data were cleaned by correcting typographical errors. Species-, month-, year- and season-specific data were aggregated and summarized by using the “aggregate” and “summary” function in the R 3.3.1[41] “*stats*” and “*base*” packages, respectively.

#### Spatial analysis

Global spatial clustering and the local indicators of spatial association (LISA) for fascioliasis cases in each year were estimated by Moran’s I [42] and LISA [43], respectively (Spatial Statistics. ArcGIS 10.3.1, Environmental System Research Institute, USA). Hotspots of fascioliasis in each year were identified using the Getis-Ord Gi* statistic in ArcGIS 10.3.1 [44]. In addition, a method of geostatistical prediction (Kriging) was used to create risk maps for each year to interpolate and predict fascioliasis cases at unmeasured locations using the observed cases at surrounding locations [45].

#### Spatial regression

A Bangladesh district shape file containing all data was imported into GeoDa 1.8.14 [46]. A spatial weight file of districts was created, based on Euclidean distance between district centroids. Initially univariable spatial regression analysis was performed using log-transformed cases as the response variable. Variables significantly (*P* < 0.10) associated with log-transformed cases in univariable regression were included in multi-variable spatial regression modelling. Multicollinearity among these potential explanatory variables was assessed by pair-wise Pearson correlation tests in R 3. 3.1. A pair of explanatory variable was considered collinear if their correlation coefficient was ≥ 0.70 [47]. In this case, the variable with a lower Akaike’s Information Criterion (AIC) was selected for multivariable spatial regression modelling. Initially, a classic (aspatial ordinary least square estimation) multivariable regression model was fit and spatial dependence diagnostics were then used to select the type of spatial model to subsequently fit to the data. A stepwise manual backward elimination method was applied to select the final model.

#### Space-time scanning

Space-time scanning was performed using SatScan Version 9.4.4 (http://www.satscan.org). A retrospective Poisson model was used to search, detect and test for significance of fascioliasis space-time clusters [48] in domestic ruminants in Bangladesh. The search was performed using circular spatial moving windows up to 10% of the population at-risk and temporal windows up to 6 months duration. A cluster in space and time was defined when there were more cases observed (*O*) within the scanning window than expected (*E*), and its statistical significance was evaluated using the log likelihood ratio statistic. The corresponding *P*-value was obtained via Monte Carlo simulations (*n* = 999). Significant clusters were visualized using ArcGIS 10.3.1 (Environmental System Research Institute, USA).

## Results

### Descriptive statistics

Fascioliasis cases were reported from 469 sub-districts (64 districts) in 2011, 461 sub-districts (63 districts) in 2012 and 454 sub-districts (62 districts) in 2013. A total of 1,723,971 cases of fascioliasis and 4,433 deaths due to fascioliasis were recorded during the three-year study period. The overall median (1^st^ quartile–3^rd^ quartile) prevalence of fascioliasis per ten thousand domestic ruminants per district in Bangladesh was100 (77–161) in three-year study period. The total number of fascioliasis cases and deaths, respectively, were 601,308 and 1,609 in 2011; 506,429 and 1,271 in 2012; 616,234 and 1,553 in 2013. The median (1^st^ quartile–3^rd^ quartile) number of cases of fascioliasis per district were 6,916 (4,719–12,146) in 2011; 6,382 (4,440–8,168) in 2012; and 8,943 (4,413–12,698) in 2013. The median (1^st^ quartile–3^rd^ quartile) number of deaths due to fascioliasis per district were 7 (0–25), 5.5 (0–32.3) and 6.5 (0–25.1) in 2011, 2012 and 2013, respectively. The highest proportion of fascioliasis cases (67.5%) was recorded in cattle and the highest case fatality (33) per ten thousand animals was recorded in goats (Table 1). Table 2 presents the monthly and seasonal distribution of fascioliasis in domestic ruminants; this varied from 5.7% in February to 10.3% in June. The proportion of fascioliasis cases was the highest (28.7%) in post-monsoon and the lowest (20.8%) in winter seasons.

**Table 1:** Species-specific distribution of fascioliasis cases and estimated case-fatality in domestic ruminants based on passive surveillance data reported from 64 districts in Bangladesh during 2011–2013

Species	Cases	Proportion (%) and 95% CI	Deaths	Case fatality^a^
Cattle	1,164,560	67.55 (67.48–67.62)	2,885	25
Goats	424,314	24.61 (24.55–24.68)	1,402	33
Buffalo	88,924	5.15 (5.12–5.19)	107	12
Sheep	46,173	2.67 (2.65–2.70)	39	8
Total	1,723,971		4,433	26

*Abbreviation*: *CI* confidence interval

^a^Per 10,000 at-risk

**Table 2:** Monthly distribution of fascioliasis in domestic ruminants based on passive surveillance data reported from 64 districts in Bangladesh during 2011–2013

Month	Cases	% (95% CI)
December	145,403	8.4
January	115,058	6.7
February	98,848	5.7
Winter (December-February)	359,309	20.84 (20.78–20.90)
March	100,472	5.8
April	134,348	7.8
May	160,317	9.3
Pre-monsoon (March-May)	395,137	22.92 (22.86–22.98)
June	176,992	10.3
July	142,807	8.3
August	155,013	8.9
Monsoon (June–August)	474,719	27.54 (27.47–27.60)
September	165,271	9.6
October	161,210	9.4
November	168,232	9.8
Post-monsoon (September–November)	494,806	28.70 (28.63–28.77)
Total	1,723,971	100

*Abbreviation*: *CI* confidence interval

### Global and local spatial clustering, time-space clusters and risk prediction

**Fig. 1:**
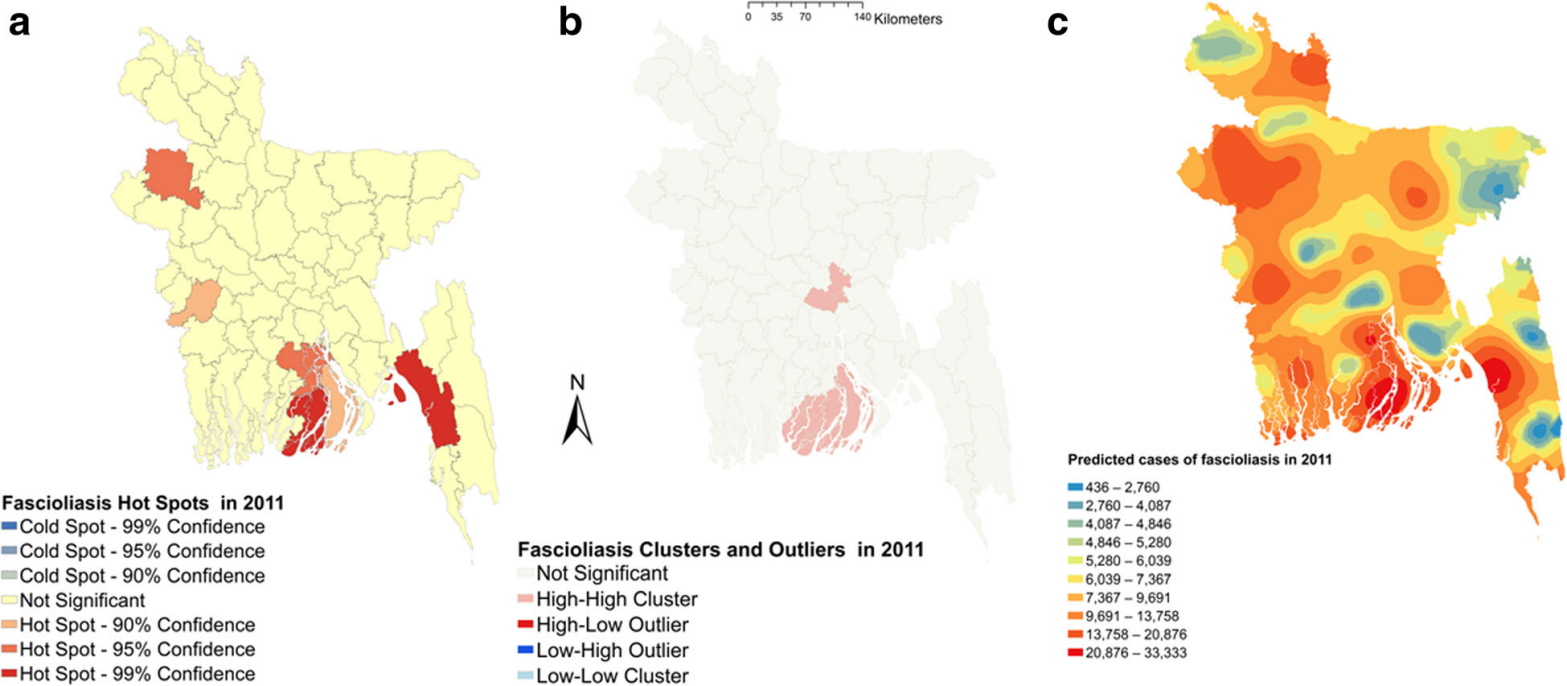
Fascioliasis hot spots, clusters and outliers and risk map in 2011. **a** Hot spots. **b** Clusters. **c** Risk map

**Fig. 2:**
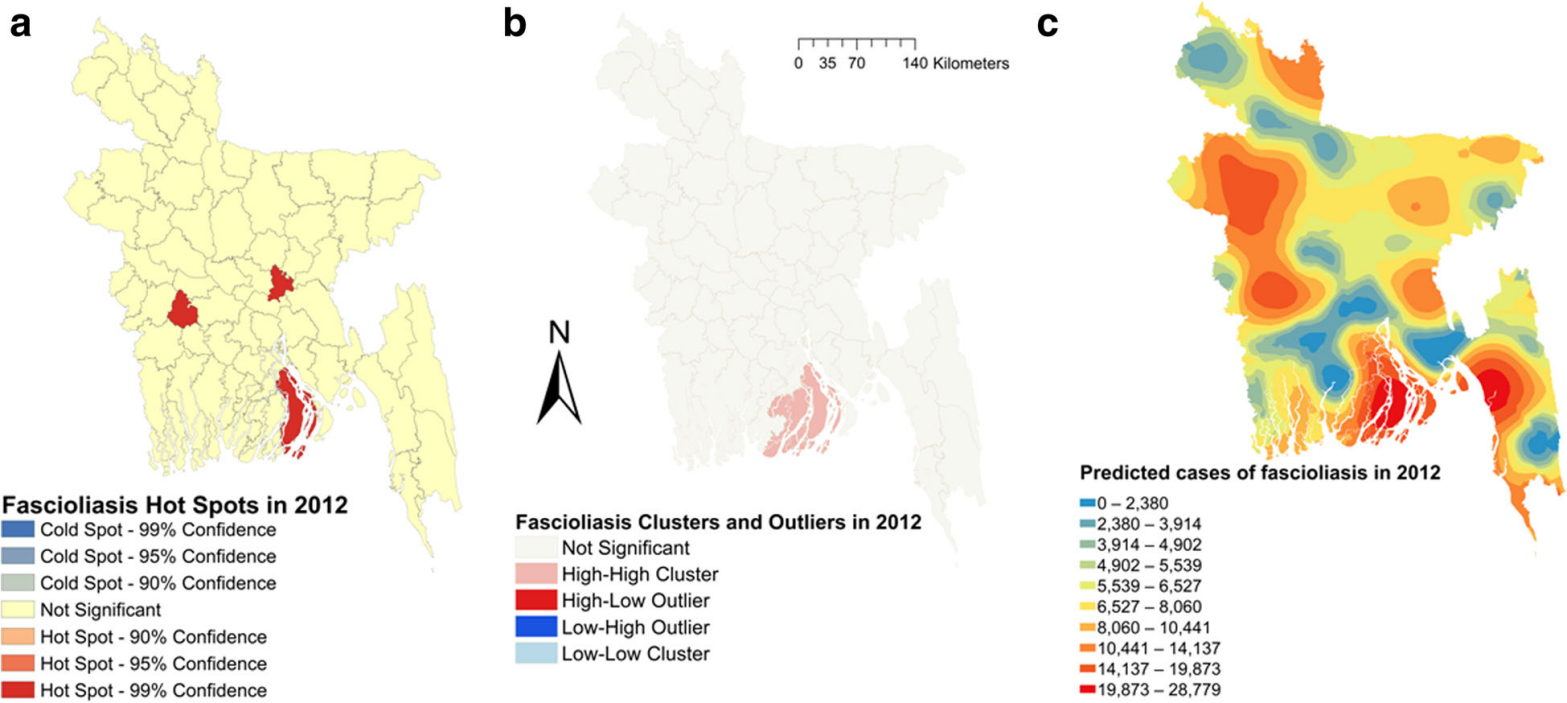
Fascioliasis hot spots, clusters and outliers and risk map in 2012. **a** Hot spots. **b** Clusters. **c** Risk map

**Fig. 3:**
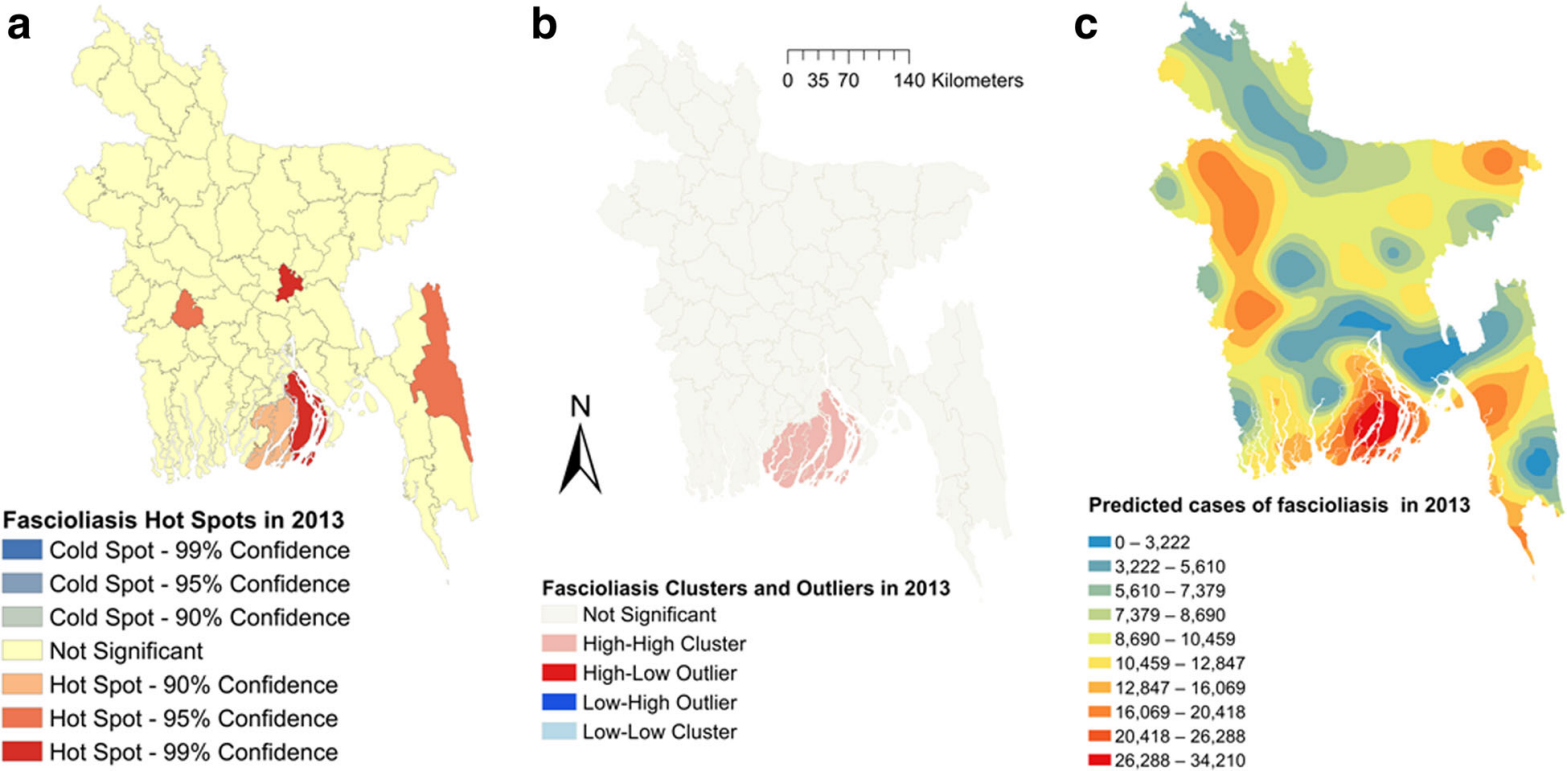
Fascioliasis hot spots, clusters and outliers and risk map in 2013. **a** Hot spots. **b** Clusters. **c** Risk map

**Fig. 4:**
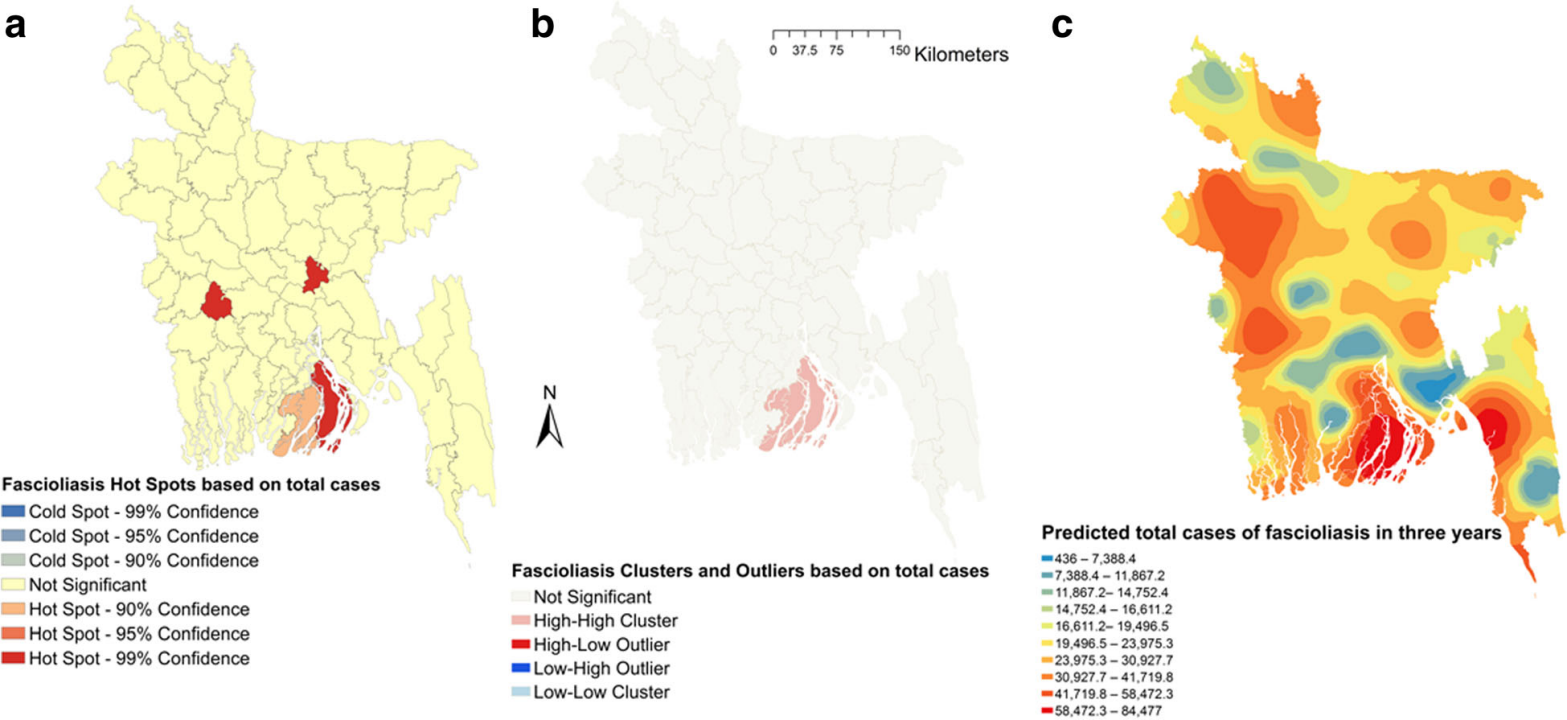
Fascioliasis hot spots, clusters and outliers and risk map based on total cases. **a** Hot spots. **b** Clusters. **c** Risk map

Moran’s I were estimated to be 0.164 (*Z*-score = 2.04, *P* = 0.04), 0.144 (*Z*-score = 1.82, *P* = 0.06) and 0.183 (*Z*-score = 2.27, *P* = 0.02), respectively, in 2011, 2012 and 2013, indicating strong global clustering of fascioliasis cases. Fig. 1 shows hotspots, clusters and outliers and predicted maps of fascioliasis cases in 2011. A total of six hotspots were detected in 2011 (Fig. 1a). Only three hotspots were identified in 2012 (Fig. 2a); among them one was common with 2011. In 2013, five hotspots were detected (Fig. 3a) of which one was common in all three years but three were common between 2012 and 2013 and two were common between 2011 and 2013. Based on total number of cases, four hotspots were identified; of these two, three and all were common in 2011, 2012 and 2013, respectively (Fig. 4a).

Two high-high (high numbers of cases in a district with high numbers of cases in surrounding districts) local clusters were detected in 2011 (Fig. 1b). Only one high-high cluster was found in 2012 and 2013 (Figs. 2b, 3b) which was also common with that of 2011. Based on the number of total fascioliasis cases, only one high-high cluster was detected which was common with 2011, 2012 and 2013 (Fig. 4b).

Fascioliasis case prediction maps were consistent with the hotspot and cluster maps in each of the respective years. The locations of the highest number of predicted cases were in south, south-east and mid-west parts of Bangladesh in each year and all three years together (Figs. 1c, 2c, 3c and 4c).

### Risk factors

**Fig. 5:**
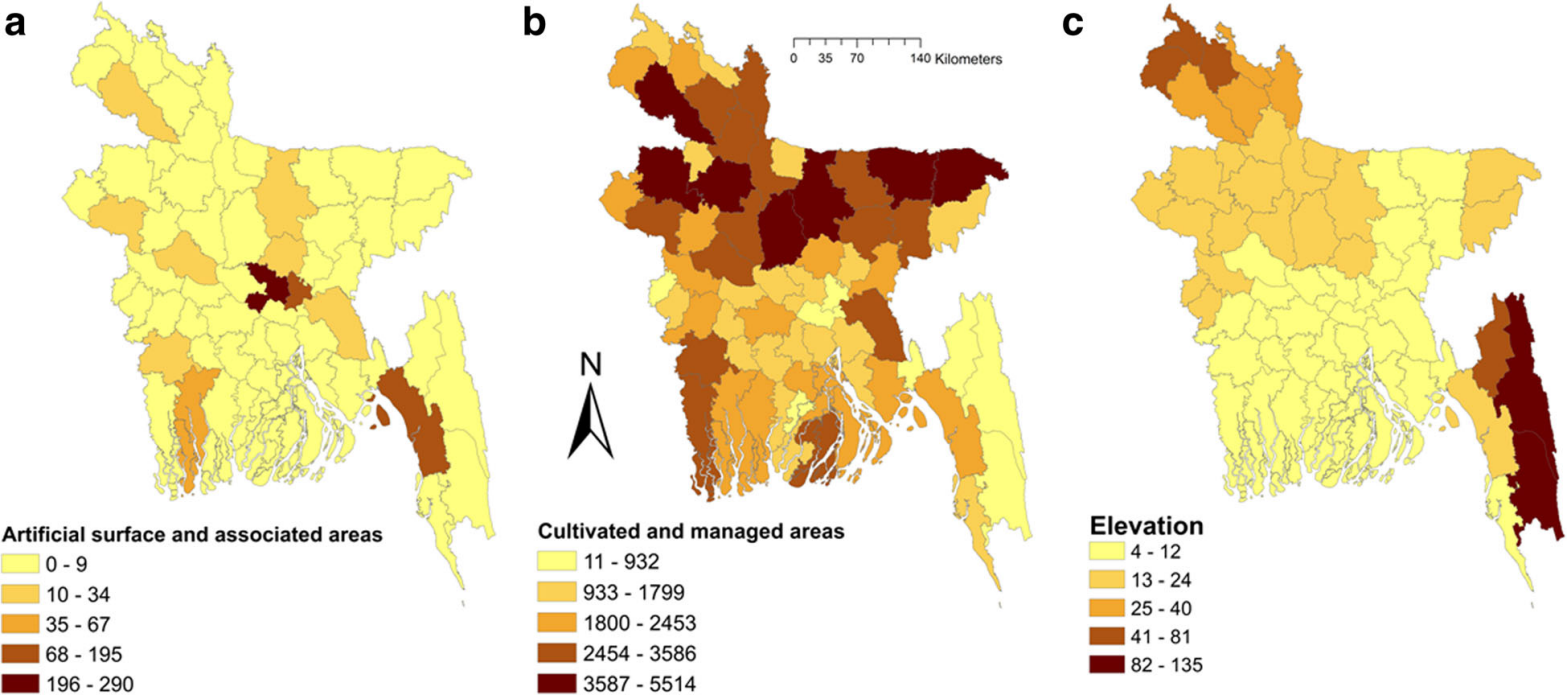
Map of Bangladesh showing significant land cover characteristics associated with fascioliasis in domestic ruminants. **a** Artificial surface and associated areas. **b** Cultivated and managed areas. **c** Elevation

**Fig. 6:**
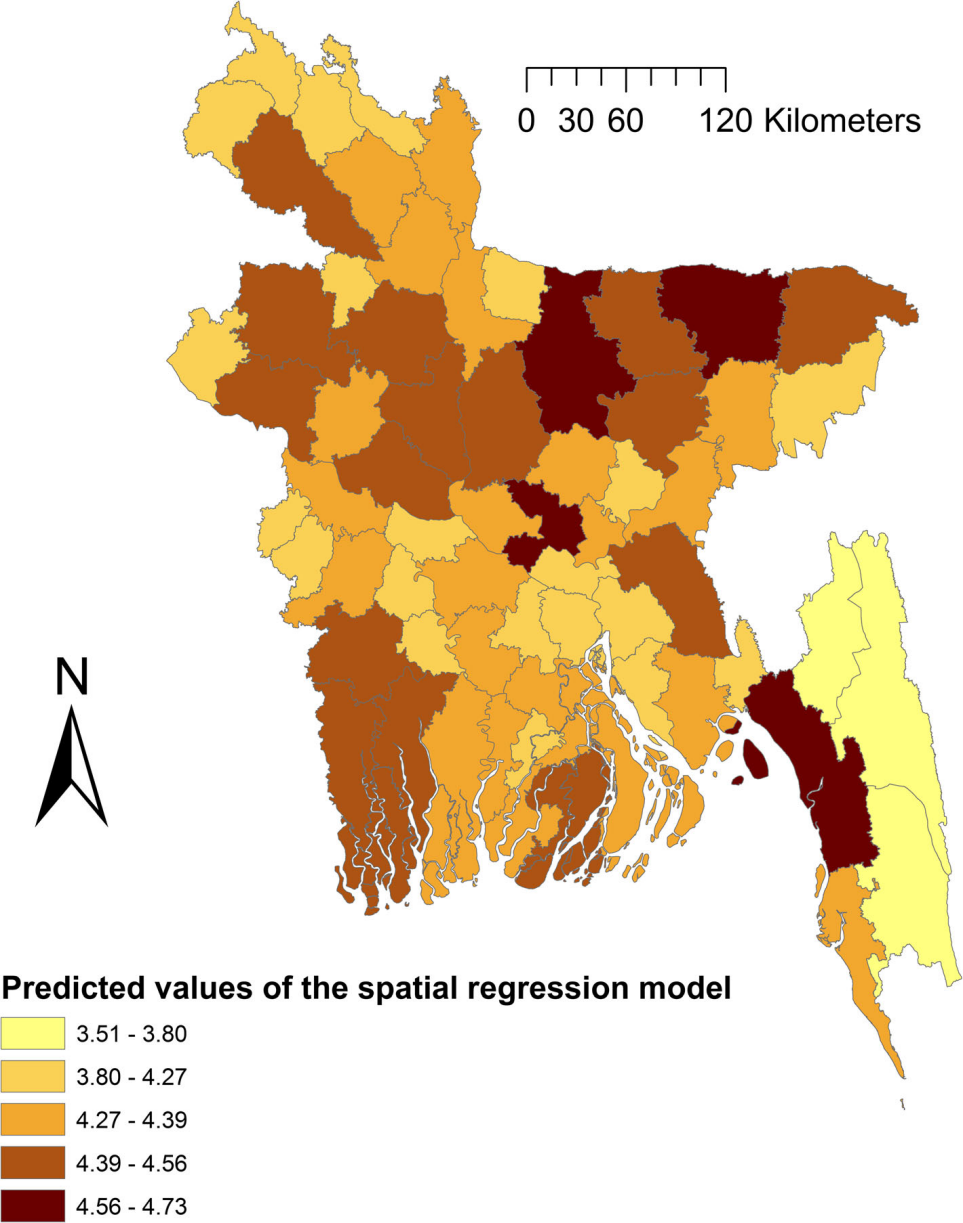
Map of Bangladesh showing predicted fascioliasis cases (log of total cases in three years) in domestic ruminants from the spatial regression model

In univariable spatial regression analysis, seasonal precipitation and temperature, elevation and several land cover classes were significantly associated with fascioliasis in domestic ruminants (Table 3). Only maximum likelihood lag test was significant in classic spatial regression indicating that a maximum likelihood spatial lag model should be used for the multivariable model. Cultivated and managed areas (*P* < 0.001) and artificial surface and associated areas (*P* = 0.04) were positively - but elevation (*P* = 0.003) was negatively - associated with fascioliasis in domestic ruminants in Bangladesh (Table 4). Fig. 4 shows maps of these three risk factors for fascioliasis in domestic ruminants in Bangladesh. Twenty one out of 64 districts in Bangladesh have high levels of cultivation (Fig. 5b). Only five districts have higher elevation (>40–135 m). Predicted fascioliasis cases (log of total cases in three years) in domestic ruminants from the spatial regression model is shown in Fig. 6.

**Table 3:** Explanatory variables associated with log-transferred fascioliasis cases in domestic ruminants in Bangladesh in univariable spatial regression analysis

Variables	Category	Coefficient	SE	*P*-value
Precipitation	Winter	-5.91	0.006	0.99
	Pre-monsoon	-0.0001	0.0003	0.59
	Monsoon	-0.0003	0.000	<0.01
	Post-monsoon	-0.001	0.005	0.04
Temperature	Winter	-0.072	0.05	0.16
	Pre-monsoon	0.037	0.05	0.45
	Monsoon	0.26	0.07	<0.01
	Post-monsoon	0.097	0.07	0.38
Elevation	−	-0.008	0.002	<0.01
Area of in-land water bodies	−	0.000	0.000	0.24
Length of river	−	-0.002	0.002	0.24
Land cover	Tree-cover, broadleaved, evergreen	-0.003	0.001	<0.01
	Tree-cover, broadleaved, deciduous, closed	-0.004	0.002	0.10
	Tree-cover, regularly flooded, saline water	0.00006	0.0001	0.64
	Mosaic: tree-cover and other natural vegetation	-0.0005	0.001	0.62
	Tree-cover, burnt	-0.0002	0.000	<0.01
	Shrub-cover, closed-open, evergreen	-0.0003	0.0003	0.36
	Shrub-cover, closed-open, deciduous	-0.1134	0.17	0.51
	Cultivated and managed areas	0.00013	0.000	<0.01
	Mosaic: cropland, tree-cover, other natural vegetation	-0.0003	0.0001	0.03
	Artificial surface and associated areas	0.0016	0.001	0.08
	Water bodies	0.00001	0.0003	0.72

*Abbreviation*: *SE* standard error

**Table 4:** Potential risk factors for fascioliasis risk in domestic ruminants in Bangladesh identified in the maximum likelihood spatial lag regression model

Risk factors	Estimate	SE	*P*-value
Elevation	−0.00472	0.00157	0.003
Cultivated and managed areas	0.00012	0.00003	0.0003
Artificial surface and associated areas	0.00156	0.00076	0.04

*Abbreviation*: *SE* standard error

### Space-time clusters

**Fig. 7:**
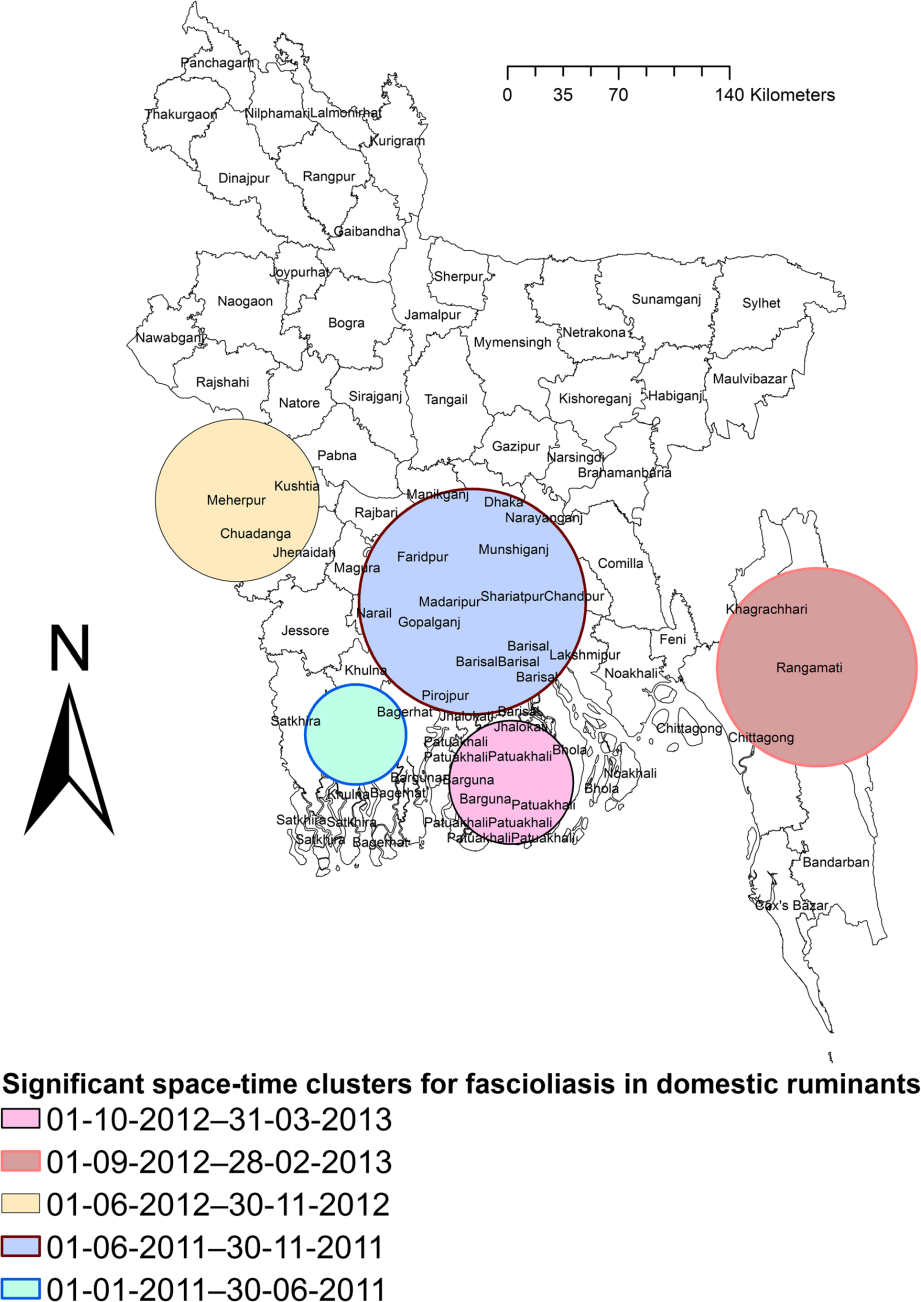
Map of Bangladesh showing significant space-time clusters of fascioliasis in domestic ruminants

Five space-time clusters in different areas were identified, including 22 districts in five divisions (Table 5). The radius of clusters varied from 31.7 to 71.4 km. The significant space-time clusters are shown in Fig. 7.

**Table 5:** Significant clusters of fascioliasis cases in domestic ruminants reported from 64 districts in Bangladesh between January 2011 and December 2013

District	Radius (km)	Observed ÷ Expected	LLR	Maximum spatial window (% study area)	Maximum temporal window (months)	Time period	*P*-value
Barguna, Bhola, Patuakhali	39.1	4.8	37792.1	10	6	01-10-2012–31-03-2013	0.001
Chittagong, Khagrachhari, Rangamati	62.7	2.5	9381.4	10	6	01-09-2012–28-02-2013	0.001
Chuadanga, Jhenaidah, Kushtia, Meherpur	51.5	1.7	4123.6	10	6	01-06-2012–30-11-2012	0.001
Barisal, Chandpur, Dhaka, Faridpur, Gopalganj, Madaripur, Munshiganj, Narail, Narayanganj, Shariatpur	71.4	2.0	9963.5	10	6	01-06-2011–30-11-2011	0.001
Khulna, Bagerhat	31.7	2.7	6129.2	10	6	01-01-2011–30-06-2011	0.001

*Abbreviation*: *LLR* Log likelihood ratio

## Discussion

To our knowledge, we have described for the first-time hotspots, clusters in space and time and risk factors for fascioliasis in domestic ruminants based on national passive surveillance data in Bangladesh. Hotspots and clusters were identified, and the distribution of fascioliasis in domestic ruminants in Bangladesh was found to be associated with elevation and land use characteristics. The findings suggest that targeted control programs can provide benefits.

More than 1.7 million fascioliasis cases in cattle, goats, sheep and buffalo were confirmed in all districts of Bangladesh during the three-year study period. The actual number of fascioliasis cases is expected to be much higher than reported because very few farmers (approximately 10%) have access to an Upazila Veterinary Hospital and therefore many clinical and especially subclinical cases remain undiagnosed in the population and underreported. Due to fascioliasis about 4–6% [29, 30] of goat-livers are reported to be condemned in Bangladesh. The cost due to condemnation of goat-livers has been estimated to be US$ 115 per thousand livers [30]. The condemnation of livers in other species and the other losses due to fascioliasis in domestic ruminant have not yet been studied in Bangladesh. The major economic losses due to fascioliasis are due to reduced milk yield (a reduction of 3–15% has been estimated) in addition to some minor losses from reduced meat production [49]. Khan et al. [50] estimated that fascioliasis causes US$0.33 and US$0.32 losses per animal per day in cattle and buffaloes, respectively, due to decreased milk yield. Treating milking animals with an effective flukicide has been estimated to increase profit 3.9times the cost of treatment [51]. Estimating the costs and benefits of a fascioliasis control strategy is valuable as a decision support tool.

The occurrence of fascioliasis in cattle (67.5%) was relatively higher than in other ruminants (2.8–24.5%). This difference might be due to more grazing by cattle compared to goats and sheep. Sheep and goats also have a selective feeding habit which might lead to less exposure to infective stages than cattle and buffaloes. Fewer reports of fascioliasis in buffaloes might be due to underreporting because this species is generally reared in low-lying rural and coastal areas and usually away from urban areas where Upazilla Veterinary hospitals are located. The intermediate host of *F. gigantica* is much more aquatic than that of *F. hepatica* [3] and only *F. gigantica* is prevalent in Bangladesh. As buffaloes inhabit wetter areas the prevalence of fascioliasis is expected to be higher than other ruminant species [50–52]. Previous published reports from Bangladesh have also reported similarly higher prevalence in cattle and buffalo than goats and sheep [14, 22, 28, 33]. However, the mortality was relatively higher in goats (33 per 10,000) than other species (8–25 per 10,000). Mortality usually occurs due to migration of immature flukes in the liver parenchyma leading to severe hemorrhage and thereby death. Liver damage due to immature *Fasciola gigantica* also predisposes to black disease (*Clostridium novyi* infection) and thus mortality. Black disease is more common in small ruminants than large ruminants [53].

Cultivated and managed areas of Bangladesh was found to be significantly associated with fascioliasis in domestic ruminants. Cultivation with irrigation creates a favorable environment for the vector snail which has been reported to be associated with high fascioliasis risk [54–56]. The 21 districts in Bangladesh which have a high level of cultivation should be prioritised in fascioliasis control programs. The use of cattle and buffalo for tilling land has been gradually replaced by power tillage in Bangladesh [57]. This is positive in terms of fascioliasis control due to reduced contamination of pasture or paddy fields by cow dung. However, where animals are still used for tillage, they should be treated (following the strategy described below) before using them in the field. Cow dung as manure should be used after composting or used as a source of bio-gas to kill *Fasciola* eggs [58]. Farmers, especially in high-risk areas, should be regularly educated by divisional or district veterinary offices about the transmission dynamics of fascioliasis. Educating farmers will build awareness about the impact of the disease and motivate them to apply control measures in a sustainable manner [59].

Artificial surfaces and associated areas of Bangladesh were also significantly associated with fascioliasis in domestic ruminants. Artificial areas do not have any direct link with the fascioliasis risk in ruminants. Rather, these areas represent urban areas which are commonly surrounded by large populations of livestock. Dhaka, Narayanganj and Chittagong districts have the largest areas of artificial surfaces, where a lot of large dairy farms are also situated [60]. Large dairy farms contain more crossbred animals which are more prone to fascioliasis than indigenous animals [59]. Thus, areas surrounding concentrated human populations should also be targeted in fascioliasis control programs. Finally, the risk of fascioliasis was significantly lower in areas of higher elevation. Only *F. gigantica* is present in Bangladesh, and it is known to be more prevalent in low-elevation areas of Asia and Africa [56, 61]. The effect of elevation might be via reduced survival of the vector of fascioliasis, or the distribution of susceptible livestock species, or both.

Five significant space-time clusters - based on the total number of cases per district - in different areas of Bangladesh were detected. These clusters were also consistent with purely spatial clusters identified in each year separately. In these areas, there is a high level of cultivation and intensive dairy cattle and buffalo farming, which might be the reasons for such clustering. The temporal distribution of the clusters was June to November and September to March in most cases. The clusters included monsoon and post-monsoon seasons, which have been found to be significantly associated with fascioliasis [62]. The highest prevalence of fascioliasis was recorded in June (10.3%) and then in November (9.8%). Based on the temporal distribution of the clusters and monthly prevalence of fascioliasis, we recommend that farmers who are able should treat their animals twice per year (June and November) but poor farmers should treat their animals at least once in June.

The strength of this study is that we have analyzed a large amount of data reported in a national passive surveillance system to identify clusters, hotspots and potential risk factors for fascioliasis in Bangladesh. This study will assist with the allocation of scarce resources for the treatment and control of fascioliasis in high risk areas. This study also has some limitations. The data on animal-level demographic and herd-level management variables and soil types as risk factors were not available, impeding further analysis. Although the data covered the whole of Bangladesh, the study might have diagnostic (subclinical cases are not reported) and reporting (only surrounding 10% of farmers have access to Upazilla Veterinary Hospitals) biases. Moreover, case data were only available at the district level (coarse resolution), rather than having the exact geographical coordinates of the farms.

## Conclusions

Fascioliasis in domestic ruminants is endemic in Bangladesh but disease hotspots and clusters in space and time exist. Cultivated and managed areas, areas of lower elevation, identified disease hotspots and clusters and areas with the highest risk of fascioliasis should be prioritised for treatment, research, extension work and other control measures in Bangladesh and in other similar geo-climatic zones throughout the world. A follow up study that focuses on herds in hotspots and clusters at a finer spatial and temporal resolution would provide additional information about risk factors for fascioliasis in Bangladesh. Developing control programs specifically for herds in these areas might produce the greatest payoff in terms of reduced disease occurrence and increased production. In addition, understanding how farmers in these areas manage the burden of fascioliasis might provide insights that can be employed in control programs elsewhere. It is important to continue monitoring endemic diseases such as fascioliasis to detect changes in distribution and to assess the benefits of control programs. The sub-district veterinary hospitals system in Bangladesh plays a vital role in monitoring endemic diseases.

## Abbreviations

AIC: Akaike’s Information Criterion
DLS: Department of Livestock Services
LISA: Local indicator of spatial association
